# European Population of *Pectobacterium punjabense*: Genomic Diversity, Tuber Maceration Capacity and a Detection Tool for This Rarely Occurring Potato Pathogen

**DOI:** 10.3390/microorganisms9040781

**Published:** 2021-04-08

**Authors:** Jérémy Cigna, Angélique Laurent, Malgorzata Waleron, Krzysztof Waleron, Pauline Dewaegeneire, Jan van der Wolf, Didier Andrivon, Denis Faure, Valérie Hélias

**Affiliations:** 1 French Federation of Seed Potato Growers (FN3PT-inov3PT), 43-45 Rue de Naples, 75008 Paris, France; angelique.laurent@inov3pt.fr (A.L.); pauline.dewaegeneire@inov3pt.fr (P.D.); 2 Institute for Integrative Biology of the Cell, Université Paris-Saclay, CEA, CNRS, 91198 Gif-sur-Yvette, France; denis.faure@i2bc.paris-saclay.fr; 3 IGEPP, Agrocampus Ouest, INRAe, University Rennes 1, F-35653 Le Rheu, France; didier.andrivon@inrae.fr; 4 Laboratory of Plant Protection and Biotechnology, Intercollegiate Faculty of Biotechnology, University of Gdansk and Medical University of Gdansk, Abrahama 58, 80-307 Gdansk, Poland; malgorzata.waleron@biotech.ug.edu.pl; 5 Department of Pharmaceutical Microbiology, Faculty of Pharmacy, Medical University of Gdansk, Al. Gen. Hallera 107, 80-416 Gdansk, Poland; krzysztof.waleron@gumed.edu.pl; 6 Biointeractions and Plant Health, Wageningen University & Research, P.O. Box 16, 6700 AA Wageningen, The Netherlands; jan.vanderwolf@wur.nl

**Keywords:** soft rot enterobacteria, *Pectobacterium punjabense*, genomic diversity, detection qPCR TaqMan assay, maceration tuber

## Abstract

Enterobacteria belonging to the *Pectobacterium* and *Dickeya* genera are responsible for soft rot and blackleg diseases occurring in many crops around the world. Since 2016, the number of described species has more than doubled. However, some new species, such as *Pectobacterium punjabense*, are often poorly characterized, and little is known about their genomic and phenotypic variation. Here, we explored several European culture collections and identified seven strains of *P. punjabense.* All were collected from potato blackleg symptoms, sometimes from a long time ago, i.e., the IFB5596 strain isolated almost 25 years ago. We showed that this species remains rare, with less than 0.24% of *P. punjabense* strains identified among pectinolytic bacteria present in the surveyed collections. The analysis of the genomic diversity revealed the non-clonal character of *P. punjabense* species. Furthermore, the strains showed aggressiveness differences. Finally, a qPCR Taqman assay was developed for rapid and specific strain characterization and for use in diagnostic programs.

## 1. Introduction

Bacteria belonging to the *Pectobacterium* and *Dickeya* genera are causal agents of soft rots in a wide variety of host plants [1]. The agricultural and economic impact ranks these pathogens classified within the group of soft rot *Pectobacteriaceae* (SRP) [2] among the most studied phytopathogenic bacteria [3]. They produce extracellular enzymes degrading plant cell walls [4,5] causing the breakdown of plant tissues. Several of the SRP can generate blackleg symptoms, characterized by a blackening of the stem base [6]. The losses can be significant [7] and, to date, chemical, physical or biocontrol applications have shown limited efficacy. Therefore, prophylactic methods such seed sanitation and breeding potato cultivars to improve natural resistance are still highly recommended [8].

Since the wide-scale use of next generation sequencing (NGS) for strain characterization, it has become clear that the genetic diversity of SRP is very high [9]. Analysis of NGS data resulted in the elevation of different subspecies to the species level [10] and also in the description of new species. As a consequence, the number of species belonging to these two genera has increased from five *Pectobacterium* spp. and seven *Dickeya* spp. before 2016 to 18 *Pectobacterium* spp. and 12 *Dickeya* spp. until now [11,12]. The NGS analysis further resulted in the revision and refining of the taxonomic position of strains maintained in laboratory or reference collections, sometimes for a long time [10,13]. The isolates were collected from different backgrounds, including diseased plant samples and surface waters [14,15,16,17,18,19,20].

Historically, the isolates collected in temperate regions from blackleg-diseased plants were identified as *Erwinia atroseptica, Erwinia carotovora* subsp. *wasabiae* and *Erwinia chrysanthemi* [21,22,23]. On the basis of the current taxonomy, *P. atrosepticum*, *P. brasiliense, P. parmentieri, P. versatile*, *P. polaris* and *P. carotovorum* are the most prevalent *Pectobacterium* species responsible for soft rot and/or blackleg diseases [24,25,26,27,28,29,30]. It should be noted, however, that the ability of pectinolytic bacteria to grow as well as develop blackleg and soft rot symptoms depends on many environmental factors such as temperature and humidity, in a species-dependent way [31]. Concerning *Dickeya* spp., *D. dianthicola* and the clonal pathogen *D. solani* are still the only two species isolated from blackleg symptoms in European potato fields [32,33,34].

The ability to detect all blackleg causing strains is of high importance. Molecular assays have been developed already for specific detection of many *Pectobacterium* and *Dickeya* species [35,36,37,38,39,40,41,42,43,44]. Nevertheless, detection methods are lacking for some of the more recently described species such as *P. polaris* [15], *P. versatile* [10] or *P. punjabense* [45].

Our study focused on *P. punjabense* species. The type strain *P. punjabense* SS95^T^ was isolated in 2017 from blackleg symptoms sampled in a Pakistani potato field. The average nucleotide identity and in silico DNA–DNA hybridization values allowed one to distinguish this strain as belonging to a new species, taxonomically close to *P. parmentieri* and *P. wasabiae*. In addition, other genomic analyzes showed that several genes of *P. punjabense* SS95^T^ involved in siderophore transport and metabolite uptake are absent in *P. parmentieri* and *P. wasabiae* genomes [45]. Since this initial description, there are no records of the pathogen from other countries. We investigated the distribution, the genetic diversity and phenotypic variation of *Pectobacterium punjabense* in Europe by the analysis of strains deposited in culture collections. We also describe the development of a qPCR TaqMan assay for specific detection of *P. punjabense*.

## 2. Materials and Methods

### 2.1. Bacterial Strains

In order to assess the presence of *P. punjabense* in European potato soft rot *Pectobacteriaceae* collections, strains of the French RNS collection from inov3PT, the Dutch IPO collection from Wageningen University and Research and the Polish IFB collection from the Intercollegiate Faculty of Biotechnology were screened by sequencing of the housekeeping genes *gapA* and *dnaX* [46,47] or *gyrA, rpoA*, *rpoS* and *recA* for the Polish collection [14,48,49]. Four strains, namely, P9A19a, RNS08.28, IPO3715 and IFB5596 (Table 1), all originating from blackleg symptoms and isolated on CVP medium [50], showed *gapA* and *dnaX* sequences similar to those of the *P. punjabense* SS95 type strain. These strains, stored in glycerol at −80 °C, were plated on Tryptone Yeast extract (TY) rich medium and were used in all following steps of this work. Seven type strains of other *Pectobacterium* species, namely, *P. polonicum* DPMP315^T^, *P. wasabiae* CFBP3304^T^, *P. parmentieri* RNS08.42.1a^T^, *P. betavasculorum* NCPPB2795^T^, *P. zantedeschiae* 9M^T^, *P. peruviense* IFB5232^T^ and *P. atrosepticum* CFBP1526^T^, were also used as reference in genomic and phenotypic comparative assays.

### 2.2. Genetic and Genomic Diversity

#### 2.2.1. Genomic Resource

The draft genomes of the four *P. punjabense* candidates were sequenced using a MiSeq Paired-End Illumina technology. Then, paired-end reads of each strain were assembled using the de novo assembly tool of CLC genomics workbench 10.1.1 (https://digitalinsights.qiagen.com accessed on 12 February 2021). The draft genome sequences were deposited in GenBank to which accession numbers have been assigned. The genome sequences representative of the seven *Pectobacterium* species closest to *P. punjabense*, as well as those of *P. punjabense* SS95^T^, were used as references in the comparative analyses (Table 1).

#### 2.2.2. Multi-Locus Sequence Analysis (MLSA)

The sequences of the fourteen housekeeping genes (*acnA*, *fusA*, *gapA*, *glyA*, *groEL*, *gyrB*, *mdh*, *mtlD*, *purA*, *recA*, *rplB*, *rpoD*, *rpoS*, *secY*) of *P. punjabense* candidates and reference type strains, recovered from the corresponding genome sequences, were concatenated, aligned and used to build a phylogenetic tree using the maximum composite likelihood method with MEGA X [51]. Bootstrap values were calculated from 1000 replicate iterations.

#### 2.2.3. Average Nucleotide Identity (ANI), In Silico DNA–DNA Hybridization (DDH) and Single Nucleotide Polymorphism (SNP) Analysis

The average nucleotide identity (ANI) calculator of the EzBioCloud server [52] was used for genome comparison and to confirm the taxonomic assignation of each *P. punjabense* candidate genome. In addition, the Genome-to-Genome Distance Calculator of the Leibniz Institute DSMZ [53] was used to calculate the DNA–DNA hybridization (DDH) value in order to obtain a second robust parameter for each genome comparison. Then, read mapping of each *P. punjabense* candidate to the *P. punjabense* SS95^T^ reference genome was performed. Finally, the variant detection tool of CLC genomics workbench 10.1.1 (https://digitalinsights.qiagen.com accessed on 12 February 2021) was used to estimate the number of SNPs for each *P. punjabense* candidate, with the *P. punjabense* SS95^T^ genome as a reference.

#### 2.2.4. BRIG Analysis

A sisual comparison of genome homology was performed using BRIG (BLAST Ring Image Generator) [54]. *P. punjabense* SS95^T^ was used as the reference genome and was compared to the other genomes of *P. punjabense*. The analysis was performed with the default settings.

### 2.3. Core Protein Phylogeny

A core phylogenetic analysis conducted from the protein sequences deduced from the concatenated core genes shared by all the genomes was compared with the clustering obtained in the multi-locus sequence analysis (MLSA). The phylogenomic analysis was performed with the PhyloPhlAn computational pipeline (https://huttenhower.sph.harvard.edu/phylophlan accessed on 18 January 2021), which uses the most conserved universal proteins from full proteomes to extract the phylogenetic signal [55]. The maximum likelihood tree was built based on 399 protein sequences. The core protein analysis was performed using the genomic sequences from the type and reference strains of the most closely related members of the genus *Pectobacterium*.

### 2.4. Carbon Source Utilization Profiles

*P. punjabense* strains (IFB5596 and 139A2, both co-isolated from the same sample in 1996, as well as RNS0828a, P9A19a and IPO3715) and strains of other closely related species such as *P. atrosepticum* (SCRI1043, ICMP1526^T^), *P. betavasculorum* (CFBP2122^T,^ CFBP5271), *P. peruviense* (IFB5232^T^, IFB9229), *P. parmentieri* (SCC3193, IFB5322), *P. wasabiae* (CFBP3304^T^, CFBP3308) and *P. zantedeschiae* (9M^T^ and 2M) were analyzed for the usage of different carbon sources using GEN III plates, and the results were compared with BIOLOG utilities. The assays were performed according to the manufacturers’ instructions.

### 2.5. Fatty Acid Methyl Esters (FAME) Composition

The whole-cell derived fatty acids were isolated directly from bacteria growing on TSA plates for 2 days at 28 °C. After saponification, methylation, extraction and washing according to the procedure described by Sasser et al. [56], the obtained fatty acid methyl esters (FAME) were analyzed using a gas chromatography-FID detector (Agilent 7820A) and an Ultra 2-HP capillary column (cross-linked 5% phenyl methyl silicone, 25 m, 0.22 mm id and 0.33 μm film thickness). A standard No. 1200-A solution (MIDI Inc., Newark, DE, USA) was used to calibrate the GC. Qualitative and quantitative analyses of the fatty acids were performed using the TSBA library (ver. 6.2B) and Sherlock Microbial Identification System software (MIDI Inc., Newark, DE, USA).

### 2.6. Tuber Maceration Assay

Assays were conducted in order to compare the tuber maceration capacity of *P. punjabense* to that of other *Pectobacterium* reference strains. In addition to *P. punjabense* P9A19a, RNS08.28, IPO3715 and SS95^T^, three *P. punjabense* isolates RNS16-153-1A, RNS18-61 and RNS18-78 were also used in the tuber maceration assay. These three isolates were collected in 2016 or 2018 from blackleg symptoms during field surveys in France and were identified as *P. punjabense* based on *dnaX* and *gapA* housekeeping gene sequences (Figure S1) in the course of this work. The tuber assay was adapted from previous works, as described in Blin et al. [34]. Inoculum suspensions of each of the tested strains were prepared from overnight cultures in TY broth and calibrated at 10^8^ cfu/mL. Two pipette tips containing 10 µL inoculum suspension each were driven into the flesh of surface-disinfected potato tubers (cv. Bintje). Eight tubers per strain were thus inoculated for a total of sixteen inoculation points per strain. A negative control with 10 µL of NaCl 0.8% was used.

Inoculated potato tubers were incubated at 25 °C in the dark in humid chambers with water-saturated air. After five days, tubers were cut across inoculation points and symptom severity was scored based on a six-class visual scale according to rot extension. To each class, a coefficient was assigned whose value increased with symptom severity (0, 0.2, 0.4, 0.6, 0.8 and 1). A pathological index (from 0 to 100) representative of the aggressiveness of each strain was calculated as follows:

(1) $$Pathological\;index=\;\frac{\sum (number\;of\;tubers\;per\;class\;\times \;class\;coefficient)}{Total\;of\;tubers}\times 100$$

Firstly, the data were analyzed using a Kruskal–Wallis non parametric rank test. Secondly, a pairwise post hoc Tukey test was performed to discriminate strains if significant differences were detected by the Kruskal–Wallis rank test.

### 2.7. qPCR TaqMan Assay

In order to find “specific regions” present in *P. punjabense* and absent in strains of the three closest species *P. polonicum*, *P. parmentieri* and *P. wasabiae*, successive Illumina-read mapping was performed with the CLC genomics workbench 10.1.1. In detail, Illumina reads of *P. punjabense* RNS08.28 were successively mapped (length fraction = 0.5; similarity = 0.8) on the five assembled *P. punjabense* genomes. Mapping reads were collected and then successively mapped on *P. parmentieri* RNS08.42.1a^T^, *P. parmentieri* WPP163, *P. parmentieri* SCC3193, *P. parmentieri* IFB5408, *P. parmentieri* IFB5427, *P. parmentieri* IFB5432, *P. parmentieri* IFB5441, *P. parmentieri* IFB5485, *P. parmentieri* IFB5486, *P. parmentieri* IFB5604, *P. parmentieri* IFB5619, *P. wasabiae* CFBP3304^T^ and *P. polonicum* DPMP315^T^ public genomes. Only the non-mapping reads were retained and de novo assembled to generate *P. punjabense*-specific contigs (minimal cut-off fixed at 2 kb).

Using these *P. punjabense* “specific regions”, primers and probes were designed using Primer3 [57]. In silico specificity of candidates was tested by a local blast on all the *Pectobacterium* genomes of the study, as well as with the Standard Nucleotide BLAST on the NCBI Nucleotide collection database. A qPCR TaqMan assay specific for *P. punjabense* was developed on a LightCycler96 Instrument real-time PCR system (Roche Life Science). The amplicon has an expected size of 217 bp and is located in a gene coding for a hypothetical protein. Amplification reactions were performed with 10 µL of FastStart Essential DNA Probes Master 2x (reference 06402682001, Roche Life Science), 3 µL of 3.33 µM primers *P. punjabense* 2b-F TCCTTCAGCCAGAGAACCAG, 3 µL of 3.33 µM primers *P. punjabense* 2b-R AACAACAATACCGGCAAGTGG, 2 µL of 2 µM probe *P. punjabense* 2b TGCAGGCCTTGTAACTCCGCT labelled with a 5′ reporter dye (FAM-6-carboxyfluorescein (FAM)) and a 3′-end quencher dye (BHQ1), synthesized by Eurofins Genomics (Ebersberg, Germany). Finally, 2 µL of template DNA was added to a final volume of 20 µL. Cycling parameters were 95 °C for 10 min, 40 cycles of 95 °C for 10 s and 60 °C for 1 min. Taqman assays were performed on a large number of strains to test the specificity of the tool. Pure DNA samples were prepared using the MasterPure™ Complete DNA kit (Lucigen) and diluted at 1 ng/µL. A serial dilution of the *P. punjabense* SS95^T^ extracted DNA ranging from 2 ng to 2.5 fg was used to determine the sensitivity of the Taqman assay. Each quantity was tested in triplicate.

## 3. Results

### 3.1. Identification and Diversity of P. punjabense Candidates

**Figure 1:**
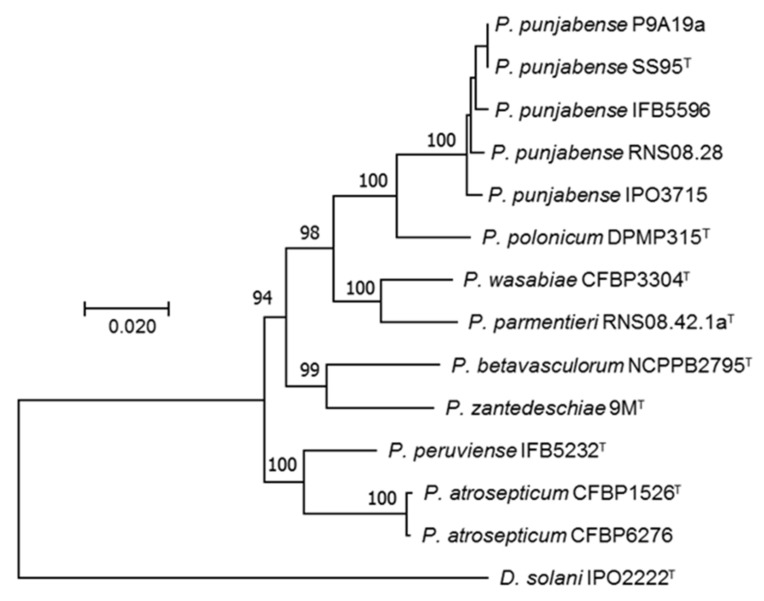
Multi-locus sequence analysis of *P. punjabense* strains and the most closely related species of the genus *Pectobacterium.* Maximum likelihood tree built with 14 housekeeping genes (*acnA*, *fusA*, *gapA*, *glyA*, *groEL*, *gyrB*, *mdh*, *mtlD*, *purA*, *recA*, *rplB*, *rpoD*, *rpoS*, *secY*) using MEGA X [51]. *D. solani* IPO2222 (CP015137) was used as an outgroup. Bootstrap values were calculated from 1000 replicates.

**Figure 2:**
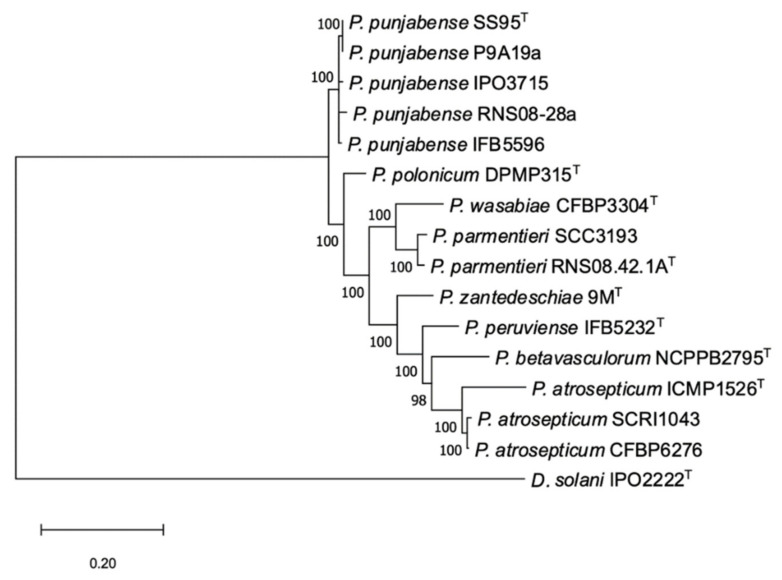
The phylogenomic analysis of *P. punjabense* strains and the most closely related species of the genus *Pectobacterium*, based on 399 most conserved universal proteins. The maximum likelihood tree was constructed using the PhyloPhlAn computational pipeline (https://huttenhower.sph.harvard.edu/phylophlan accessed on 18 January 2021). The gene sequences of *D. solani* IPO2222 (CP015137) were used as an outgroup. Bootstrap values were calculated from 1000 replicates.

The MLSA phylogenic tree including strains tentatively identified as *P. punjabense* derived from European collections and type strains from phylogenetically related *Pectobacterium* species was built from the concatenated sequences of fourteen housekeeping genes extracted from genomes. All *P. punjabense* candidates grouped together with the type strain *P. punjabense* SS95. The resulting cluster presented a robust bootstrap value (Figure 1). The core protein phylogeny confirmed the status of P9A19a, RNS08.28, IPO3715 and IFB5596 as *P. punjabense* strains (Figure 2).

The ANI and DDH values were calculated to consolidate the assignation of the European isolates P9A19a, RNS08.28, IPO3715 and IFB5596 to *P. punjabense* species. When the ANI and DDH values were calculated between *P. punjabense* genomes (Table 2), all values were higher than the accepted cut-off value for species delineation: 95 for ANI values and 70 for DDH [58,59]. The ANI and DDH values of *P. punjabense* isolates ranged from 98.6 to 100 and from 88.6 to 100, respectively. These infra-specific variations highlight the non-clonal character of *P. punjabense* strains. The ANI and DDH parameters also confirmed that *P. polonicum* DPMP315 is closely related to *P. punjabense* strains, with a mean ANI value calculated at 93.9 and a DDH of 54.9. Then, *P. parmentieri* RNS08.42.1a and *P. wasabiae* CFBP3304 are the two other strains closest to the *P. punjabense* strains with ANI values ranging between 91.0 and 91.5 and DDH values between 43.3 and 44.4.

In addition, a variant detection analysis to estimate the number of SNPs between each *P. punjabense* strain and the type strain SS95 yielded approximately 35,000 SNPs for RNS08.28 and IPO3715. For IFB5596, approximately 33,000 SNPs were found. Surprisingly, no SNP was found for P9A19a, suggesting a clonal origin of these Pakistani and French isolates. Finally, pairwise ANI values, SNP analysis and also BRIG analysis used for genome comparison (Figure S2) confirmed that the four European *P. punjabense* strains are genetically different.

### 3.2. Physiological and Structural Characteristics

**Figure 3:**
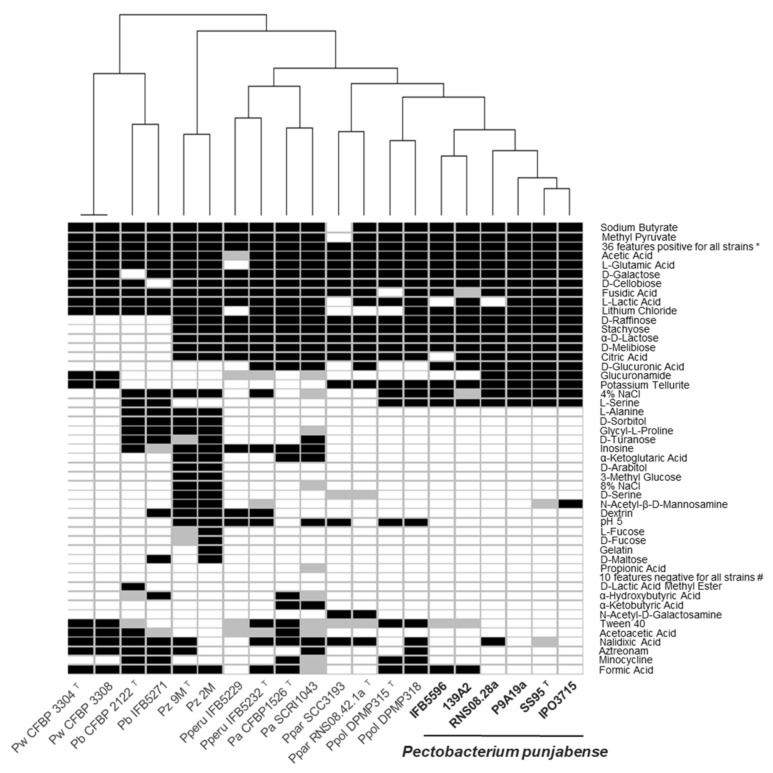
Results of the BIOLOG assay with the strains panel of *P. punjabense* and other closely related species.

The physiological and biochemical features of all the *P. punjabense* strains were very similar in the BIOLOG GEN III plates system (Figure 3). None of the single carbon sources alone could discriminate *P. punjabense* from the other *Pectobacterium* species. Noticeably, all *P. punjabense* strains assimilated D-glucuronic acid and L-serine as the sole carbon source, while most other strains tested could not. Only *P. punjabense*, *P. parmentieri* and *P. polonicum* grew in the presence of the inorganic compound potassium tellurite. *P. punjabense* strains displayed a high sensitivity for different antibiotic compounds such as nalidixic acid, aztreonam, minocycline and formic acid compared with the other species. In particular, the two strains of *P. betavasculorum* were able to grow in the presence of each of these four antibiotic compounds.

Fatty acid methyl esterase (FAME) analysis revealed the presence of in total 28 fatty acids in the biomass of strains analyzed. Among these, 22 were detected in *P. punjabense* strains, from which 18 were present in all of the analyzed *P*. *punjabense* strains. Six out of 28 detected fatty acids were absent in this species. The most prevalent are the following fatty acids: 12:0, 16:0, 17:1 ω8c, 17:0, 12:0 aldehyde/unknown 10.928/16:1 iso I/14:0 3OH, 16:1 ω7c/16:1 ω6c, 18:1 ω7c/18:1 ω6c (Table S1). Overall, the fatty acid contents of *P. punjabense* strains were homogenous and similar to those of other *Pectobacterium* strains. However, fatty acids 17:0 cyclo, 19:0 iso and 19:0 cyclo ω8c were absent in the *P. punjabense* strains but present in the most closely related *P. polonicum* DPMP315 strain.

### 3.3. Occurrence of P. Punjabense in Collections

The three bacterial collections used in our study gather SRP collected over many years from different sources, although strains from blackleg=diseased plants are overrepresented. For collection, a CVP medium has been used that allows the isolation of all SRP with largely the same efficiency [50]. We therefore could make an estimate on the isolation incidence of *P. punjabense* relative to other SRP strains (Table 3). For example, over the five sampling campaigns between 2015 and 2019, 1663 strains were deposited in the RNS collection, including only four *P. punjabense* strains, all collected from different fields. In the IPO collection, among 1012 SRP characterized between 1963 and 2020, only one *P. punjabense* strain was identified. From 2031 strains sampled in Poland between 1996 and 2014 (including 1500 strains isolated in 1996), four *P. punjabense* isolates were identified, but all were isolated from the same blackleg diseased plant, and they showed identical housekeeping genes sequences.

### 3.4. Aggressiveness

**Figure 4:**
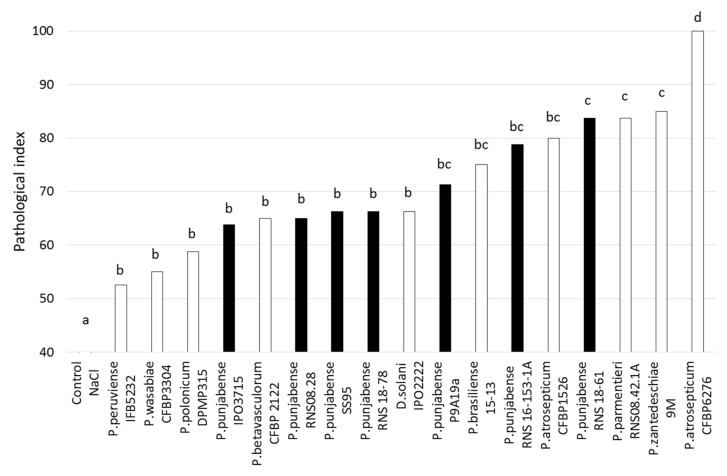
Soft rot index in a potato tuber assay comparing *P. punjabense* strains (in black) to other representative *Pectobacterium* strains (in white). Different lowercase letters above bars indicate statistically significant differences (Tukey HSD test, *p* = 0.05).

The *P. punjabense* strains varied in their tuber maceration capacity (Figure 4). For strains IPO3715, RNS08-28, SS95^T^ and RNS18-78, the pathological index was significantly lower than that of RNS 18-61, which had an index similar to that of the reference strain *P. zantedeschiae* 9M^T^, previously described as a strain with a high maceration capacity [18]. Strains P9A19a and RNS16-153-1a presented a pathological index of 71 and 79, respectively, indicating an intermediate aggressiveness level compared to the other strains of the panel. Finally, none of the *P. punjabense* strains tested showed a potential maceration capacity equivalent to the referent aggressive strain *P. atrosepticum* CFBP6276 under the conditions of the experiment.

### 3.5. Development of a qPCR TaqMan Assay Specific for P. Punjabense

The qPCR Taqman 2b assay gave a strong signal (Ct values ranging from 17.58 to 20.29) with DNA extracted from the eight *P. punjabense* strains (the five strains used for the genomic work and the three additional isolates included in the pathogenicity assay), but not with DNA from 59 different *Pectobacterium* and *Dickeya* species, all calibrated at 1 ng/µL (Table 4).

To determine the threshold level, a ten-fold serial dilution of *P. punjabense* SS95^T^ DNA ranging from 2 ng to 2.5 fg was tested. The detection threshold of the qPCR Taqman 2b was obtained at 20 fg of *P. punjabense* DNA, with a mean detection value of 34.52 Ct and a standard deviation of 0.98 (Table 5). The analysis of raw curves generated by the LightCycler96 Instrument real-time PCR system allowed fixing the efficiency of the qPCR reaction at 1.99, which is very close to the expected theoretical value of 2.00.

## 4. Discussion

The recent development of next generation sequencing technologies considerably improved knowledge of bacterial genomics including those of the genus *Pectobacterium*. It became evident that this genus is highly diverse, and NGS analysis also led to improved identification of strains in culture collections and the description of several new *Pectobacterium* species.

This is the case of *P. punjabense* species, whose type strain SS95 was isolated from blackleg diseased potatoes in Pakistan in 2017. Until now, SS95 was the only *P. punjabense* strain described [45]. By exploring strains of different European work collections on the basis of sequences of housekeeping genes homology, we uncovered seven strains assignable to *P. punjabense*, and the genomes of four of them were sequenced. MLSA (Figure 1) and core phylogenic protein analysis (Figure 2), as well as ANI and DDH genomic parameters (Table 2), confirmed that these four new strains indeed belong to *P. punjabense*. The *P. punjabense* strains could not be distinguished from other species tested on the basis of the fatty acid profile (Table S1).

The strains RNS08-28 and P9A19a were isolated in France in 2008 and 2015, respectively, while strain IPO3715 was isolated in the Netherlands in 2013. The oldest strain in our sample, IFB5596, isolated in 1996, comes from a Polish potato field. These data provide interesting information on the prevalence of this species. First, *P. punjabense* is present in Europe and is not limited to Pakistan, where it was initially described. Furthermore, the Polish strain IFB5596 indicates that *P. punjabense* has been present in Europe for at least 25 years but has always gone unnoticed due to a lack of specific detection tools. All strains identified as *P. punjabense* were isolated from blackleg diseased plants, but they remain very rare among isolated strains; in the three collections analyzed, the isolation frequency of *P. punjabense* strains varied from 0.05 to 0.24% of the total number of SRP strains isolated during the sampling period (Table 3).

The genomic data of the *P. punjabense* strains also provided valuable information on the diversity within this species. In particular, the ANI comparisons between *P. punjabense* strains showed values ranging from 98.6 to 100.0 (Table 2), which is comparable to those observed in the closely related species *P. parmentieri*, with values ranging from 98.92 to 99.97 [60]. Surprisingly, the two *P. punjabense* strains SS95^T^ and P9A19a showed exactly the same genomic sequences (ANI of 100.0; DDH of 100.0; no SNP found) although they were isolated in two distant countries and in two different years. In addition, no seed potatoes of the cultivar from which strain P9A19a was isolated are exported to Pakistan. There is no information on the cultivar from which the Pakistani strain SS95 was isolated, but there is no trade of Pakistani seed potatoes into Europe. As a result, no available data link these two strains to a possible common event. If we exclude the comparison between SS95^T^ and P9A19a, the average ANI for the other *P. punjabense* comparisons is 98.8 ± 0.1 and the mean DDH value is 89.3 ± 0.8. The relatively high genomic diversity is consistent with the finding that the pathogen has been present for a long time in Europe [61]. This hypothesis is reinforced by the high number of SNPs observed when comparing the different strains of *P. punjabense* identified to the type strain (more than 30,000). Therefore, the situation of *P. punjabense* over time is clearly distinct from that of the emergence of the clonal pathogen *D. solani* in European potato fields [33], where less than 100 SNPs were observed between two *D. solani* genomes [62].

With our current knowledge, the question of the *P. punjabense* reservoir remains unresolved. Indeed, the low frequency of *P. punjabense* found among isolates collected from blackleg symptoms, combined with the genomic diversity observed, suggests that *P. punjabense* is present in other niches beside potato plants, but no reference is available to support this hypothesis. In recent surveys of water from French rivers, no *P. punjabense* have been isolated [63], while *P. versatile* [10] or *P. aquaticum* [17] were recovered. *P. punjabense* was also not found within the French Collection for Plant-associated Bacteria (CIRM-CFBP), which includes over 265 *Pectobacterium* strains isolated from 1944 to 2020 and which represent isolates from a high diversity of plants [28]. Furthermore, the low incidence of *P. punjabense* strains sampled from blackleg-diseased plants in comparison to other species of *Pectobacterium,* such as *P. brasiliense*, *P. parmentieri* or *P. atrosepticum*, indicates that the potato plant is probably not the preferred host for this species.

The *P. punjabense* isolates tested were able to macerate potato tubers, indicating that they can cause soft rot during storage of tubers. For most strains investigated in this study, the ability of *P. punjabense* to cause potato blackleg has not been determined yet, but in a field experiment with vacuum-infiltrated seed tubers, strain IPO3715 did not result in diseased plants, whereas in the same experiment *P. brasiliense* showed a high disease incidence of 80% [64]. Therefore, further investigations are needed to assess the host range of *P. punjabense.*

Testing phenotypic characteristics using BIOLOG plates revealed that the *P. punjabense* strains have a lower capacity to grow in the presence of most of the antibiotic compounds tested, compared to the other pectinolytic bacteria of the panel (Figure 3). These data suggest that in complex environments, such as field soil where microbial competition is intense and phytosanitary products are widely used, *P. punjabense* could be less competitive [65]. This could partly explain the small number of strains collected in fields.

To date, no specific molecular tools are available to detect *P. punjabense* [37,38]. They showed a negative or a weak signal around the expected value at 434 bp and additional nonspecific bands with the generic PCR Y1-Y2 assay widely used to detect *Pectobacterium* sp. [35]. In order to clarify this, the alignment of the pectate lyase sequences of the strains of the panel revealed six mismatches between the nucleotides of the Y1 primer and the corresponding pectate lyase region in *P. punjabense* strains (Figure S3); this probably explains the negative PCR result observed with the Y1-Y2 primers. This information could also partly explain why *P. punjabense* strains remained poorly identified, sometimes for many years. In case of negative amplification obtained with the generic Y1-Y2 primers, some isolates have possibly been assimilated to contaminants. The Taqman 2b developed in this study has shown both its specificity and its sensitivity towards the *P. punjabense* species and will be helpful for rapid identification, seed testing, surveys and studies on epidemiology and disease management.

Overall, this study provides a first comprehensive look at *P. punjabense* in Europe and provides also information on its diversity. The data have shown that it has been present for almost 25 years, but the low frequency of isolation combined with the lack of specific identification tools have prevented its recognition. The Taqman 2b assay developed in this study will enable further studies in the potato ecosystem, including the capacity to cause blackleg and the host range.

## Figures and Tables

**Table 1:** *P. punjabense* strains selected for draft genome sequencing and additional representative *Pectobacterium* genomes used for genetic analysis.

Strain	Year of Isolation	Origin	Isolation Source	GenBank Accession Number
*P. punjabense* P9A19a ^1^	2015	France	Potato blackleg symptom	JADARA000000000
*P. punjabense* RNS08.28 ^1^	2008	France	Potato blackleg symptom	JADARB000000000
*P. punjabense* IPO3715 ^1^	2013	The Netherlands	Potato blackleg symptom	JADDMS000000000
*P. punjabense* IFB5596 ^1^	1996	Poland	Potato blackleg symptom	LXFY00000000
*P. punjabense* SS95^T^	2017	Pakistan	Potato blackleg symptom	PYSO00000000.1
*P. polonicum* DPMP315^T^	2016	Poland	Groundwater	RJTN00000000.1
*P. wasabiae* CFBP3304^T^	1985	Japan	Horseradish	CP015750.1
*P. parmentieri* RNS08.42.1a^T^	2008	France	Potato blackleg symptom	CP015749.1
*P. betavasculorum* NCPPB2795^T^	1972	USA	Sugar beet soft rot	JQHM00000000.1
*P. zantedeschiae* 9M^T^	2005	Poland	Calla lily tuber	NWTM00000000.1
*P. peruviense* IFB5232^T^	1970s	Peru	Potato plants	LXFV00000000.1
*P. atrosepticum* CFBP1526^T^	1957	United Kingdom	Potato blackleg symptom	ALIV00000000.1

^1^ Draft genome was sequenced and assembled in this study.

**Table 2:** Average nucleotide identity (ANI) and DNA–DNA hybridization (DDH) values for each genome comparison. Comparison between *P. punjabense* strains are marked in green.

Strain	Code	(1)	(2)	(3)	(4)	(5)	(6)	(7)	(8)	(9)	(10)	(11)	(12)	(13)	
*P. punjabense* SS95	(1)		**100.0**	**88.7**	**89.4**	**88.6**	54.6	44.1	43.3	38.3	38.5	38.6	37.3	37.3	**DDH**
*P. punjabense* P9A19a	(2)	**100.0**		**88.7**	**89.4**	**88.7**	54.6	44.2	43.3	38.3	38.5	38.6	37.3	37.3	
*P. punjabense* RNS08.28a	(3)	**98.7**	**98.7**		**91.0**	**89.5**	55.1	44.4	43.6	38.2	38.4	38.7	37.3	37.4	
*P. punjabense* IPO3715	(4)	**98.8**	**98.7**	**98.9**		**90.0**	55.1	44.4	43.7	38.4	38.6	38.8	37.3	37.5	
*P. punjabense* IFB5596	(5)	**98.7**	**98.6**	**98.8**	**98.8**		54.9	44.3	43.3	38.4	38.5	38.7	37.6	37.5	
*P. polonicum* DPMP315	(6)	93.9	93.8	93.9	93.9	93.9		43.1	42.8	38.2	38.3	38.2	37.2	37.5	
*P. parmentieri* RNS08.42.1a	(7)	91.3	91.4	91.5	91.4	91.4	91.0		54.9	39.5	39.6	39.6	37.6	37.8	
*P. wasabiae* CFBP3304	(8)	91.1	91.0	91.3	91.2	91.2	91.0	93.9		40.0	40.3	39.9	37.9	38.5	
*P. atrosepticum* CFBP1526	(9)	89.4	89.3	89.4	89.5	89.3	89.3	89.8	90.0		96.0	54.0	43.8	47.0	
*P. atrosepticum* CFBP6276	(10)	89.4	89.4	89.4	89.5	89.4	89.3	89.9	90.1	99.5		54.2	44.0	47.1	
*P. peruviense* IFB5232	(11)	89.6	89.6	89.5	89.6	89.6	89.4	89.8	90.0	93.7	93.7		44.2	47.3	
*P. zantedeschiae* 9M	(12)	89.1	89.0	89.0	89.0	89.2	89.1	89.2	89.4	91.3	91.2	91.4		44.4	
*P. betavasculorum* CFBP2122	(13)	89.0	89.0	89.1	89.1	89.0	89.0	89.1	89.4	91.9	91.9	92.1	91.4		
	**ANI**														

**Table 3:** Occurrence of *P. punjabense* strains in collections.

Collection	Isolation Period	Total Number of SRP Strains in Collection ^1^	*P. punjabense* Strains Identified ^2^	Frequency of *P. punjabense* Strains (%)
RNS	2015–2019	1663	4	0.24
IPO	1963–2020	1012	1	0.10
IFB	1996–2014	2031	1	0.05

^1^ Number of characterized soft rot *Pectobacteriaceae* (*Dickeya* and *Pectobacterium*) strains; ^2^ corresponding to different isolates (i.e., coming from different origins and/or with different housekeeping gene sequences).

**Table 4:** Specificity of the qPCR Taqman 2b for detection of *Pectobacterium punjabense* on DNA extracted from strains belonging to various *Pectobacterium* and *Dickeya* species.

Species	Strain	Isolation Source	Geographical Origin, Year of Isolation	Detection in Taqman Assay (Ct Value)	
***Pectobacterium atrosepticum***					
*P. atrosepticum*	CFBP1526^T^	*Solanum tuberosum*	UK, 1957	-	
*P. atrosepticum*	CFBP6276	*Solanum tuberosum*	France, 1999	-	
*P. atrosepticum*	CFBP1453	*Solanum lycopersicum*	France, 1973	-	
*P. atrosepticum*	CFBP1527	*Solanum tuberosum*	USA, 1973	-	
*P. atrosepticum*	CFBP5394	*Solanum tuberosum*	Algeria, 1999	-	
*P. atrosepticum*	CFBP3139	Soil	UK, 1962	-	
*P. atrosepticum*	CFBP7375	*Solanum tuberosum*	Syria, 2004	-	
***Pectobacterium parmentieri***					
*P. parmentieri*	RNS08-42-1A^T^	*Solanum tuberosum*	France, 2008	-	
*P. parmentieri*	CFBP1338	*Solanum tuberosum*	UK, 1970s	-	
*P. parmentieri*	CFBP1342	*Solanum tuberosum*	UK, 1970s	-	
*P. parmentieri*	CFBP5382	*Solanum tuberosum*	Netherlands, 1997	-	
*P. parmentieri*	SS90	*Solanum tuberosum*	Pakistan, 2017	-	
*P. parmentieri*	SCC3193	*Solanum tuberosum*	Finland, 1980s	-	
***Pectobacterium wasabiae***					
*P. wasabiae*	CFBP 3304^T^	*Eutrema wasabi*	Japan, 1985	-	
***Pectobacterium punjabense***					
*P. punjabense*	SS95^T^	*Solanum tuberosum*	Pakistan, 2017	17.58	
*P. punjabense*	IPO3715	*Solanum tuberosum*	Netherlands, 2013	17.78	
*P. punjabense*	RNS08-28	*Solanum tuberosum*	France, 2008	20.29	
*P. punjabense*	RNS16-153	*Solanum tuberosum*	France, 2016	19.19	
*P. punjabense*	RNS18-61	*Solanum tuberosum*	France, 2018	19.53	
*P. punjabense*	RNS18-78	*Solanum tuberosum*	France, 2018	19.94	
*P. punjabense*	P9A19a	*Solanum tuberosum*	France, 2015	17.63	
*P. punjabense*	IFB5596	*Solanum tuberosum*	Poland, 1996	18.61	
***Pectobacterium cacticida***					
*P. cacticida*	CFBP3628^T^	*Carnegiea gigantea*	USA, 1944	-	
*P. cacticida*	CFBP3217	*Carnegiea gigantea*	USA, 1959	-	
*P. cacticida*	CFBP3219	*Carnegiea gigantea*	USA, 1966	-	
***Pectobacterium peruviense***					
*P. peruviense*	IFB5232^T^	*Solanum tuberosum*	Peru, 1970s	-	
***Pectobacterium brasiliense***					
*P. brasiliense*	RNS13-47-1A	*Solanum tuberosum*	France, 2013	-	
*P. brasiliense*	CFBP5837	Water	Spain, 1990s	-	
***Pectobacterium carotovorum***					
*P*. *carotovorum*	ICMP 5702^T^	*Solanum tuberosum*	Denmark, 1952	-	
*P*. *carotovorum*	CFBP5374	*Solanum tuberosum*	Canada, 1994	-	
***Pectobacterium aroidearum***					
*P. aroidearum*	CFBP8168^T^	*Zantedeschia aethiopica*	South Africa, 1959	-	
***Pectobacterium odoriferum***					
*P. odoriferum*	NCPPB 3839^T^	*Cichorium intybus*	France, 1978	-	
*P. odoriferum*	CFBP5668	*Cichorium intybus*	France, 1983	-	
***Pectobacterium zantedeschiae***					
*P. zantedeschiae*	9M^T^	*Zantedeschia aethiopica*	Poland, 2005	-	
***Pectobacterium betavasculorum***					
*P. betavasculorum*	CFBP2122^T^	*Beta vulgaris*	USA, 1972	-	
***Pectobacterium polonicum***					
*P. polonicum*	DPMP315^T^	Groundwater	Poland, 2016	-	
***Pectobacterium fontis***					
*P. fontis*	M022^T^	Waterfall	Malaysia, 2013	-	
***Pectobacterium versatile***					
*P. versatile*	SS96	*Solanum tuberosum*	Pakistan, 2017	-	
*P. versatile*	S4.16.03.3F	*Solanum tuberosum*	Morocco, 2016	-	
*P. versatile*	S4.16.03.3I	*Solanum tuberosum*	Morocco, 2016	-	
*P. versatile*	RNS98-1	*Solanum tuberosum*	France, 1998	-	
***Pectobacterium polaris***					
*P. polaris*	S4.16.03.2B	*Solanum tuberosum*	Morocco, 2016	-	
*P. polaris*	SS28	*Solanum tuberosum*	Pakistan, 2017	-	
***Dickeya dianthicola***					
*D. dianthicola*	NCPPB 453^T^	*Dianthus caryophyllus*	UK, 1956	-	
*D. dianthicola*	CFBP2015	*Solanum tuberosum*	France, 1975	-	
*D. dianthicola*	CFBP1888	*Solanum tuberosum*	France, 1978	-	
*D. dianthicola*	MIE32	*Solanum tuberosum*	Israel, ?	-	
*D. dianthicola*	MIE33	*Pelargonium capitatum*	Switzerland, 1988	-	
*D. dianthicola*	MIE34	*Solanum tuberosum*	Switzerland, 2013	-	
***Dickeya dadantii***					
*D. dadantii*	CFBP3695	*Zea mays*	Cuba, 1987	-	
*D. dadantii*	3937	*Saintpaulia ionantha*	France, 1977	-	
*D. dadantii*	CFBP2051	*Dieffenbachia* sp.	USA, 1957	-	
***Dickeya solani***					
*D. solani*	IPO 2222^T^	*Solanum tuberosum*	Netherlands, 2007	-	
*D. solani*	RNS05-1-2A	*Solanum tuberosum*	France, 2005	-	
*D. solani*	Am3a	*Solanum tuberosum*	France, 2015	-	
*D. solani*	MK16	River water	UK, ?	-	
*D. solani*	RNS07-7-3B	*Solanum tuberosum*	France, 2007	-	
*D. solani*	CC3239	*Solanum tuberosum*	UK, ?	-	
***Dickeya paradisiaca***					
*D. paradisiaca*	CFBP4178^T^	*Musa paradisiaca*	Colombia, 1970	-	
***Dickeya zeae***					
*D. zeae*	CFBP3707	Water	Israel, 1986	-	
***Dickeya chrysanthemi***					
*D. chrysanthemi*	CFBP3704	*Cynara scolymus L.*	Reunion island, 1986	-	
*D. chrysanthemi*	CFBP6689	*Cichorium endivia*	France, 2002	-	
***Dickeya undicola***					
*D. undicola*	2B12^T^	Lake water	Malaysia, 2014	-	
*D. undicola*	FVG1	Fresh water	France, 2017	-	
*D. undicola*	FVG10	Fresh water	France, 2016	-	
***Dickeya fangzhongdai***					
*D. fangzhongdai*	CFBP8607^T^	*Pyrus pyrifolia*	China, 2009	-	
*D. fangzhongdai*	B16	*Phalaenopsis* sp.	Slovenia, 2010	-	

-: no amplification; Ct value: cycle threshold value, defined as the number of cycles required for the fluorescent signal to exceed the detection threshold.

**Table 5:** Sensitivity results observed with qPCR Taqman 2b on a Standard curve of *P. punjabense* SS95^T^ DNA.

DNA Concentration	Repetition	Ct Value	Ct Mean	Standard Deviation
2 ng	1	17.54	17.55	0.07
	2	17.48		
	3	17.62		
200 pg	1	20.97	21.02	0.04
	2	21.05		
	3	21.03		
20 pg	1	24.57	24.54	0.03
	2	24.53		
	3	24.51		
2 pg	1	27.87	27.82	0.05
	2	27.78		
	3	27.82		
200 fg	1	31.21	31.47	0.23
	2	31.56		
	3	31.64		
20 fg	1	33.96	34.52	0.98
	2	35.65		
	3	33.96		
10 fg	1	36.75	35.24	1.32
	2	34.64		
	3	34.33		
5 fg	1	-	ND	ND
	2	-		
	3	-		
2.5 fg	1	-	ND	ND
	2	37.16		
	3	-		
Control	1	-	ND	ND
	2	-		
	3	-		

ND: not determined. -: no amplification.

## Data Availability

Data presented in this study are available in the present manuscript and its supplementary information file.

## Supplementary Materials

The following are available online at https://www.mdpi.com/article/10.3390/microorganisms9040781/s1, Figure S1: The *gapA* molecular phylogenetic analysis for taxonomic assignation of *P. punjabense* candidates. The evolutionary history was inferred by using the maximum likelihood method. There were a total of 846 positions in the final dataset. Bootstrap values were calculated from 1000 replicate iterations. Evolutionary analyses were conducted in MEGA X, Figure S2: BLAST comparisons of four *P. punjabense* genomes sequenced against the *P. punjabense* SS95^T^ genome performed with the BRIG application, Figure S3: Y1 sequences alignment with different *Pectobacterium* strains. Nucleotide represented in grey corresponds to that of the reference sequence (primer Y1). Nucleotide in bold red represents a mismatch with that of the reference sequence, Table S1: The percentage of total amount of fatty acids detected in the cells of five *P. punjabense* strains (SS95^T^, IFB5596, RNS08.28, P9A19a, IPO3715), *P. polonicum* DPMP315^T^, *P. wasabiae* (CFBP3304^T^, CFBP3308), *P. parmentieri* (RNS08.42.1A^T^, SCC3193), *P. atrosepticum* (CFBP1526^T^, CFBP6276), *P. peruviense* (IFB5232^T^), *P. zantedeschiae* (9M^T^) and *P. betavasculorum* (CFBP2122^T^).
