# Supplementary material for: European Population of Pectobacterium punjabense: Genomic Diversity, Tuber Maceration Capacity and a Detection Tool for This Rarely Occurring Potato Pathogen
Source: Microorganisms. 2021 Apr 8;9(4):781. doi: 10.3390/microorganisms9040781 (PMC8068253; doi:10.3390/microorganisms9040781)
Supplement: Supplementary file 1 [file microorganisms-09-00781-s001.pdf]

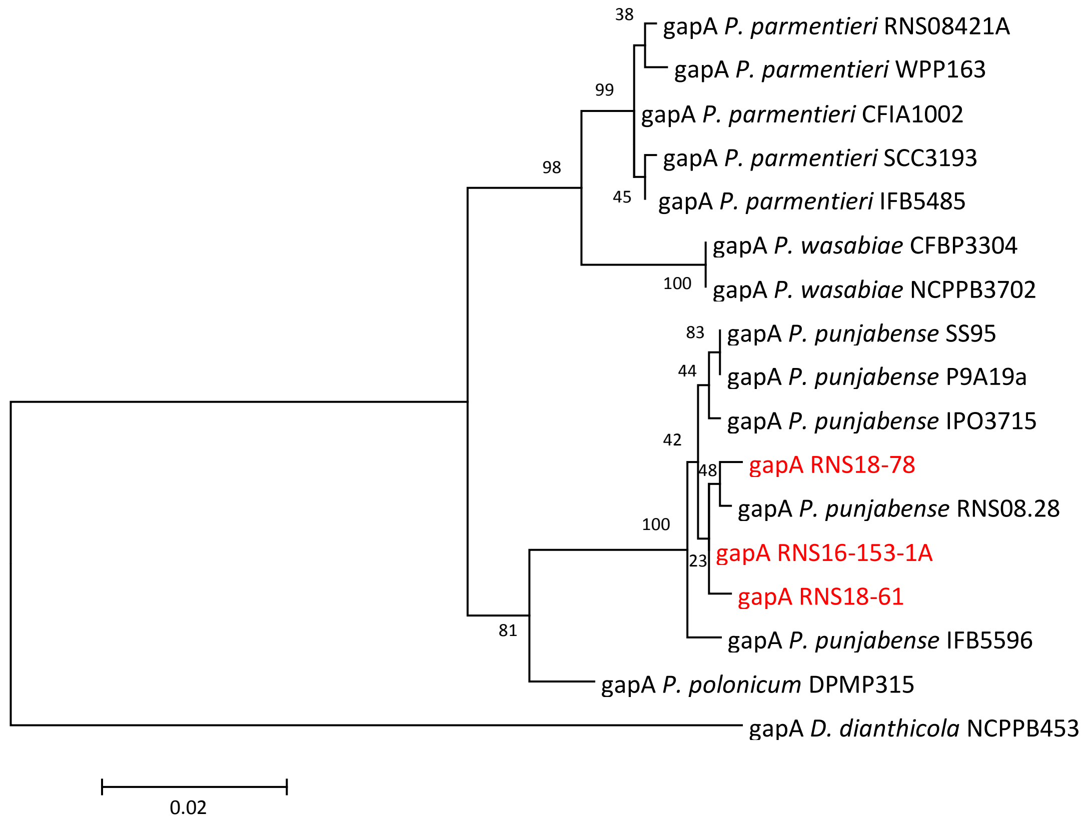

Figure S1. The gapA Molecular Phylogenetic analysis for taxonomic assignation *of P.punjabense* candidates.
The evolutionary history was inferred by using the Maximum Likelihood method. There were a total of 846 positions in the final dataset. Bootstrap values were calculated from 1000 replicate iterations. Evolutionary analyses were conducted in MEGA X.

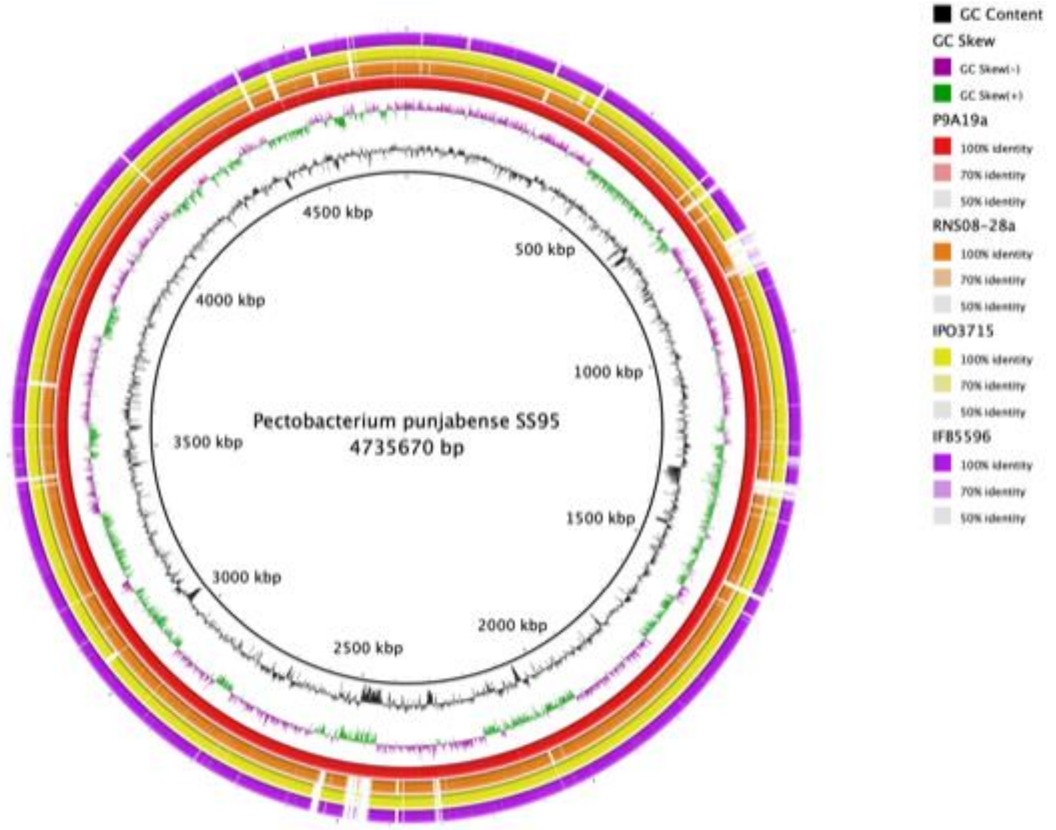

Figure S2. BLAST comparisons of 4 *P. punjabense* genomes sequenced against the *P. punjabense* SS95^{T} genome performed with the BRIG application

Primer Y1	5’	T T A C C G G A C G C C G A G C T G T G G C G T 3’
P. punjabense SS95^{T}		T **C** A C C **A** G A **T** G C **T** G A **A** C T **A** T G G C G T
P. punjabense P9A19a		T **C** A C C **A** G A **T** G C **T** G A **A** C T **A** T G G C G T
P. punjabense RNS08.28		T **C** A C C **A** G A **T** G C **T** G A **A** C T **A** T G G C G T
P. punjabense IPO3715		T **C** A C C **A** G A **T** G C **T** G A **A** C T **A** T G G C G T
P. punjabense IFB5596		T **C** A C C **A** G A **T** G C **T** G A **A** C T **A** T G G C G T
P. polonicum DPMP315^{T}		T T A C C G G A C G C C G A G C T G T G G C G T
P. versatile CFBP6051^{T}		T T A C C G G A C G C C G A G C T G T G G C G T
P. carotovorum ICMP5702^{T}		T T A C C G G A C G C C G A G C T G T G G C G T
P. brasiliense LMG21371^{T}		T **C** A C C G G A C G C C G A **A** C T G T G G C G T
P. polaris NIBIO1006^{T}		T **C** A C C G G A C G C C G A **A** C T G T G G C G T
P. peruviense IFB5232^{T}		T **C** A C C G G A C G C C G A **A** C T G T G G C G T
P. atrosepticum CFBP1526^{T}		T **C** A C C G G A C G C C G A **A** C T G T G G C G T
P. atrosepticum CFBP6276		T **C** A C C G G A C G C C G A **A** C T G T G G C G T
P. zantedeschiae 9M^{T}		T **C** A C C G G A C G C **T** G A **A** C T G T G G C G T
P. parmentieri RNS08.42.1A^{T}		T **C** A C C G G A **T** **A** C C G A G **T** T G T G G C G T
P. parmentieri WPP163		T **C** A C C G G A **T** **A** C C G A G C T G T G G C G T
P. wasabiae CFBP3304^{T}		T **C** A C C **A** G A **T** **A** C C G A G C T G T G G C G T

Figure S3. Y1 sequences alignment with different *Pectobacterium* strains. Nucleotide represented in grey corresponds to that of the reference sequence (primer Y1). Nucleotide in bold red represents a mismatch with that of the reference sequence.

Table S1. The percentage of total amount of fatty acids detected in the cells of five *P. punjabense* strains (SS95^{T}, IFB5596, RNS08.28, P9A19a, IPO3715), *P. polonicum* DPMP315^{T}, *P. wasabiae* (CFBP3304^{T}, CFBP3308), *P. parmentieri* (RNS08.42.1A^{T}, SCC3193), *P. atrosepticum* (CFBP1526^{T}, CFBP6276), *P. peruviense* (IFB5232^{T}), *P. zantedeschiae* (9M^{T}) and *P. betavasculorum* (CFBP2122^{T}).

	P. punjabense					*P. polonicum*	*P. wasabiae*		*P. parmentieri*		*P. atrosepticum*		*P. peru.*	*P. zant.*	*P. beta.*
Fatty Acid	**SS95**	**IFB5596**	**RNS08.28**	**P9A19a**	**IPO3715**	DPMP315	CFBP3304	CFBP3308	SCC3193	RNS0842	CFBP1526	CFBP6276	IFB5232	9M	CFBP2122
9:0	ND	ND	ND	0,08	ND	ND	0,13	0,175	0,11	0,13	ND	ND	0,08	0,11	0,05
10:0	0,225	0,165	0,17	0,21	0,255	ND	0,14	0,195	0,18	0,215	0,195	0,175	0,13	0,29	0,17
11:0	0,535	0,52	0,605	0,7	0,495	ND	0,79	1,235	0,81	0,89	0,39	0,205	0,54	0,725	0,615
12:1 at 11-12	ND	ND	ND	ND	ND	ND	ND	ND	ND	ND	0,14	0,13	ND	ND	ND
12:0	5,235	5,605	5,6	5,365	5,355	2,05	3,86	4,765	3,905	3,83	5,155	4,23	4,305	5,22	5,73
11:0 3OH	ND	ND	ND	ND	ND	ND	ND	ND	ND	ND	ND	ND	ND	ND	0,1
13:0	2,26	2,715	2,53	3,005	2,495	0,37	3,465	4,715	3,34	3,835	1,565	0,9	2,51	2,85	1,775
12:0 3OH	ND	ND	ND	ND	ND	ND	ND	ND	ND	ND	ND	0,25	ND	ND	0,71
14:0	1,03	1,49	1,205	1,245	1,23	6,025	1,21	0,95	1,16	1,215	141,805	1,25	1,19	1,1	0,75
15:0 anteiso	0,14	0,165	0,355	0,19	0,2	0,31	0,175	0,19	0,175	0,17	0,18	0,16	0,145	0,175	0,14
15:1 ω8c	0,49	0,99	0,87	1,13	1,285	ND	1,69	1,56	1,75	685,565	0,81	0,255	0,955	1,23	0,355
15:1 ω6c	0,34	0,18	0,09	0,17	0,21	ND	0,26	0,25	0,24	0,285	0,18	ND	0,2	0,165	0,14
16:1 ω5c	0,21	0,24	0,14	ND	0,17	ND	0,15	0,13	0,185	0,16	0,165	0,285	0,2	0,15	ND
16:0	16,025	17,58	17,015	16,06	16,545	25,405	14,845	16,305	14,7	14,485	23,96	13,585	16,465	17,49	13,09
15:0 3OH	0,76	0,825	0,755	0,925	0,84	ND	1,065	1,18	1,05	1,19	0,57	0,335	0,86	0,755	0,32
17:0 anteiso	0,155	0,165	0,29	0,19	0,18	0,35	0,14	0,16	ND	0,16	0,15	0,22	0,14	0,175	0,17
17:0 cyclo	ND	ND	ND	ND	ND	11,72	ND	ND	ND	ND	1,22	ND	ND	ND	ND
17:1 ω8c	4,465	2,025	1,98	2,62	2,61	0,5	5,375	3,815	4,779	5,49	1,935	2,035	2,835	2,49	3,56
17:1 ω6c	1,83	0,415	0,52	0,7	0,665	ND	1,74	1,095	1,48	1,735	1,21	0,525	0,73	0,835	1,33
17:00	5,17	1,73	2,505	2,23	2,435	1,505	6,68	4,96	6,085	6,63	1,765	1,22	2,13	3,165	4,705
18:1 ω9c	ND	ND	ND	0,31	ND	1,38	ND	0,18	0,45	0,28	ND	0,2	0,13	0,19	ND
18:0	0,275	ND	0,22	0,24	ND	0,42	0,22	0,17	ND	0,185	ND	0,28	0,22	0,215	0,155
19:0 iso	ND	ND	ND	ND	ND	0,54	ND	ND	ND	ND	ND	ND	ND	ND	ND
19:0 cyclo ω8c	ND	ND	ND	ND	ND	1,53	ND	ND	ND	ND	ND	ND	ND	ND	ND
Sum in Feature 1 ❶	2,6	2,695	2,995	3,51	2,39	0,74	4,265	5,825	4,145	4,61	1,39	0,805	2,455	3,19	2,765
Sum in Feature 2 ❷	7,895	8,46	8,49	8,54	8,16	8,945	6,945	7,405	7,125	6,945	9,06	8,695	8,08	8,255	7,48
Sum in Feature 3 ❸	29,38	35,365	33,295	33,17	34,375	19,38	29,29	28,26	31,04	29,085	38,44	37,975	36,4	31,22	29,35
Sum in Feature 8 ❹	21,2	18,645	20,74	19,42	20,29	20,42	17,755	16,64	17,48	16,69	10,925	26,115	19,245	20,23	26,565

The values given are expressed as a percentage. ND = not determined

Summed features contain two fatty acids that couldn't have been separated with the MIDI system:

- Summed Feature 1 ❶ 15:1 iso H/13:0 3OH/13:0 3OH
- Summed Feature 2 ❷ 12:0 aldehyde/unknown 10.928/ 16:1 iso I/14:0 3OH
- Summed Feature 3 ❸ 16:1 ω7c/16:1 ω6c
- Summed Feature 8 ❹ 18:1 ω7c/18:1 ω6c
